# Genetic differentiation that is exceptionally high and unexpectedly sensitive to geographic distance in the absence of gene flow: Insights from the genus *Eranthis* in East Asian regions

**DOI:** 10.1002/ece3.9007

**Published:** 2022-06-07

**Authors:** Ami Oh, Byoung‐Un Oh

**Affiliations:** ^1^ 34933 Department of Biology Chungbuk National University Cheongju Chungbuk Republic of Korea

**Keywords:** *Eranthis*, gene flow, genetic differentiation, geographic distance, population

## Abstract

Genetic differentiation between populations is determined by various factors, including gene flow, selection, mutation, and genetic drift. Among these, gene flow is known to counter genetic differentiation. The genus *Eranthis*, an early flowering perennial herb, can serve as a good model to study genetic differentiation and gene flow due to its easily detectable population characteristics and known reproductive strategies, which can be associated with gene flow patterns. *Eranthis* populations are typically small and geographically separated from the others. Moreover, previous studies and our own observations suggest that seed and pollen dispersal between *Eranthis* populations is highly unlikely and therefore, currently, gene flow may not be probable in this genus. Based on these premises, we hypothesized that the genetic differentiation between the *Eranthis* populations would be significant, and that the genetic differentiation would not sensitively reflect geographic distance in the absence of gene flow. To test these hypotheses, genetic differentiation, genetic distance, isolation by distance, historical gene flow, and bottlenecks were analyzed in four species of this genus. Genetic differentiation was significantly high, and in many cases, extremely high. Moreover, genetic differentiation and geographic distance were positively correlated in most cases. We provide possible explanations for these observations. First, we suggest that the combination of the marker type used in our study (chloroplast microsatellites), genetic drift, and possibly selection might have resulted in the extremely high genetic differentiation observed herein. Additionally, we provide the possibility that genetic distance reflects geographic distance through historical gene flow, or adaptation in the absence of historical gene flow. Nevertheless, our explanations can be more rigorously examined and further refined through additional observations and various population genetic analyses. In particular, we suggest that other accessible populations of the genus *Eranthis* should be included in future studies to better characterize the intriguing population dynamics of this genus.

## Graphical abstract

In our study, under the low probability of gene flow, genetic differentiation between populations was significantly high and genetic distance generally reflected geographic distance in the genus Eranthis. We suggest that significantly high level of genetic differentiation is due to the marker type used, genetic drift, and selection. We also suggest that the positive correlation between genetic distance and geographic distance might have resulted from historical gene flow or adaptation without historical gene flow.

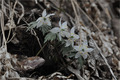

## 1 INTRODUCTION

Assessment of genetic differentiation between populations allows for the characterization of taxa of interest and is therefore of great importance in the field of population genetics. Genetic differentiation, an indicator of how much populations are genetically isolated from others, is determined by various factors including gene flow, selection, mutation, and genetic drift (Slatkin, 1987). Among these, gene flow, which is the movement of genes between populations, is a critical factor that determines genetic differentiation (Ellstrand, 1992; Slatkin, 1987). Gene flow is generally known to counter genetic differentiation (Ehrlich & Raven, 1969; Rifkin et al., 2019; Slatkin, 1987). In plant populations, gene flow occurs either by pollen or seed dispersal (Ennos, 1994). Previous studies have argued that plant gene flow via wind and insect pollination, as well as by seed dispersal, can be spatially restricted (Ehrlich & Raven, 1969; Harper, 1977; Howe & Smallwood, 1982). For instance, in wind‐pollinated *Zea mays*, only 1% of outcrossing contamination was observed in a population that was approximately 18 m from the source population (Ehrlich & Raven, 1969). As another example, in *Clarkia* and *Delphinium*, which are pollinated by insects, the distances shorter than 15m could be an effective barrier to the pollination (Roberts & Lewis, 1955). However, there are some cases for long‐distance gene flow, for example, in tree species (Burczyk et al., 2004; Dow & Ashley, 1998; Liepelt et al., 2002; Ony et al., 2020; Schuster et al., 1989).

**FIGURE 1:**
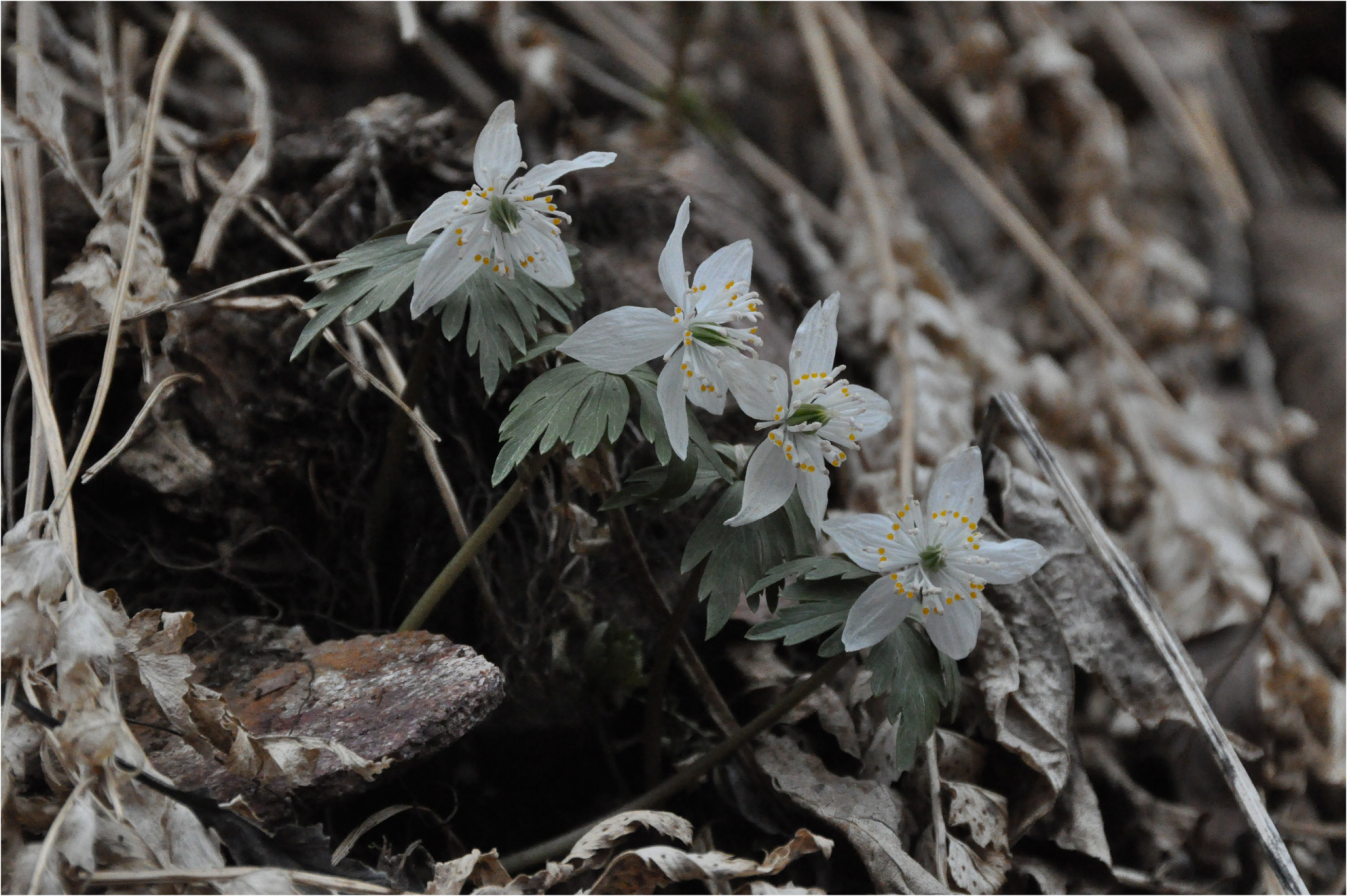
*Eranthis stellata* of Gapyeong, Republic of Korea

The genus *Eranthis* (Ranunculaceae), an early flowering perennial herb (Figure 1), provides an interesting case study for genetic differentiation and gene flow. This genus is composed of approximately 13 species, which are distributed from Europe to East Asia (Erst, Sukhorukov, et al., 2020; Erst et al., 2020b; Mabberley, 1993; Rukšāns, 2018; Tamura, 1990; Wang et al., 2001). This genus comprises relatively short plants (5–15 cm above ground) with tuberous rhizomes, palmately divided leaves and bracts, solitary flowers, and petaloid sepals (Oh & Ji, 2009; Sun et al., 1993). The life history and ecology of this genus have only been characterized in some species. For example, *Eranthis hyemalis*, which is distributed throughout Europe, is known to reproduce by vegetative reproduction using split tubers (Marcinkowski, 2002) and by sexual reproduction via insect pollination (Rysiak & Žuraw, 2011). Regarding growth conditions, *E.* *byunsanensis* in South Korea generally grows in gentle valleys with organic matter‐rich soil, and this species may be sensitive to moisture conditions (Kim et al., 2012).

In the cases of *E. byunsanensis*, *E. pungdoensis*, and *E. stellata*, which inhabit South Korea, it was observed that there were only a small number of populations in a single province of South Korea, which means that the populations of these species are rarely observed. The populations of these species, whose sizes generally range from 300 to 20,000 m^2^, do not cluster together, and therefore become geographically isolated from each other (generally isolated by 10 km~, personal observation). In addition, it has been reported that populations are often separated by forest roads or mountain trails within the same site (Kim et al., 2012). The *Eranthis* species in other regions than South Korea also show similar distributional patterns (personal observation).

Meanwhile, some reports indicate that the seeds of the genus *Eranthis* cannot disperse over long distances, and from personal observation, the dispersal distance would be only up to 1 m. The species in this genus produce rounded or sub‐globose seeds approximately 2–3 mm in diameter (Jung et al., 2010; Oh & Ji, 2009) which can only disperse through gravity and wind. Additionally, Thomson et al. (2011) found that seed dispersal distance is strongly correlated with plant height, suggesting that the seeds of the genus *Eranthis* (which is 5–15 cm tall above ground) may not disperse long distances.

Regarding insect pollination in this genus, even though insects carry pollen with relatively greater specificity than in the case of wind pollination, gene flow via this route is known to be more unlikely than originally expected (Ehrlich & Raven, 1969). For instance, bumblebees and anthophorid bees exhibit high visitation rates for target flowers, but travel only short distances between them (Rust, 1980; Kudoh and Whigham, 1997), meaning that insect‐facilitated gene flow between populations is not likely to occur in some cases. Furthermore, it has been suggested that early flowering in chilling temperatures may lead to low insect pollination in *E.* *byunsanensis* and *Megaleranthis saniculifolia*, the latter of which is morphologically and taxonomically similar to the genus *Eranthis*, resulting in low possibility of long‐distance pollen dispersal (Jeong, 2010; Kim et al., 2012; Waser, 1982). Overall, it appears that gene flow via pollination only occurs selectively in this genus. Given the observed characteristics of the populations for this genus, as well as previous literatures on gene flow, it seems very unlikely that gene flow is currently occurring between the populations of each species within this genus.

To date, only one study has characterized the population genetics of the genus *Eranthis*, which analyzed *E.* *byunsanensis* with 10 allozyme markers (So et al., 2012). In this study, considerably high genetic variation within population and little genetic differentiation among populations were observed. However, the restricted number of markers and the marker type used in the aforementioned study might have rendered less accurate conclusions regarding this species. Apart from this study, the population genetics of this genus has remained largely uncharacterized. In our study, we sought to investigate genetic differentiation and gene flow in the genus *Eranthis*, and tried to understand current and past population dynamics for this genus so that we can provide valuable fundamentals for population genetics in the genus *Eranthis*.

We hypothesized that due to the current low likelihood of gene flow between populations, as discussed above, the genetic differentiation between the populations in each species of this genus would be generally significant. Additionally, we hypothesized that genetic differentiation between the populations would not sensitively reflect the geographic distance between the populations in the absence of current gene flow.

To test these hypotheses, our study analyzed genetic differentiation, genetic distance, isolation by distance, historical gene flow, and bottlenecks in four East Asian *Eranthis* species including *Eranthis byunsanensis*, *Eranthis pungdoensis*, *Eranthis stellata*, and *Eranthis pinnatifida*. We then discussed the potential evolutionary/ecological mechanisms which can explain the intriguing population dynamics we observed in the genus *Eranthis*.

## 2 MATERIALS AND METHODS

### 2.1 Population sampling

Four *Eranthis* species for which samples were available, including *Eranthis byunsanensis*, *Eranthis pungdoensis*, *Eranthis stellate*, and *Eranthis pinnatifida*, were analyzed in this study. *E.* *byunsanensis* is endemic to Korea, with the northern limit likely being within North Korea. *E.* *pungdoensis* is found only in Pungdo, a small island in the West Sea of South Korea. *E.* *byunsanensis* and *E.* *pungdoensis* were only recently reported as new species (Oh & Ji, 2009; Sun et al., 1993). Due to their similar morphological and genetic characteristics, these two species are thought to be evolutionarily closely related (Oh & Oh, 2019). *E.* *stellata* is found in the Korean peninsula, the Jilin and Liaoning provinces of China, and some of the easternmost regions of Russia. *E.* *pinnatifida* ranges from the central to southern areas of Honshu, Japan. The morphological differences between these species are generally observed in the shapes of petals, leaves, and bracts. Nectary patterns are also different between these species.

**FIGURE 2:**
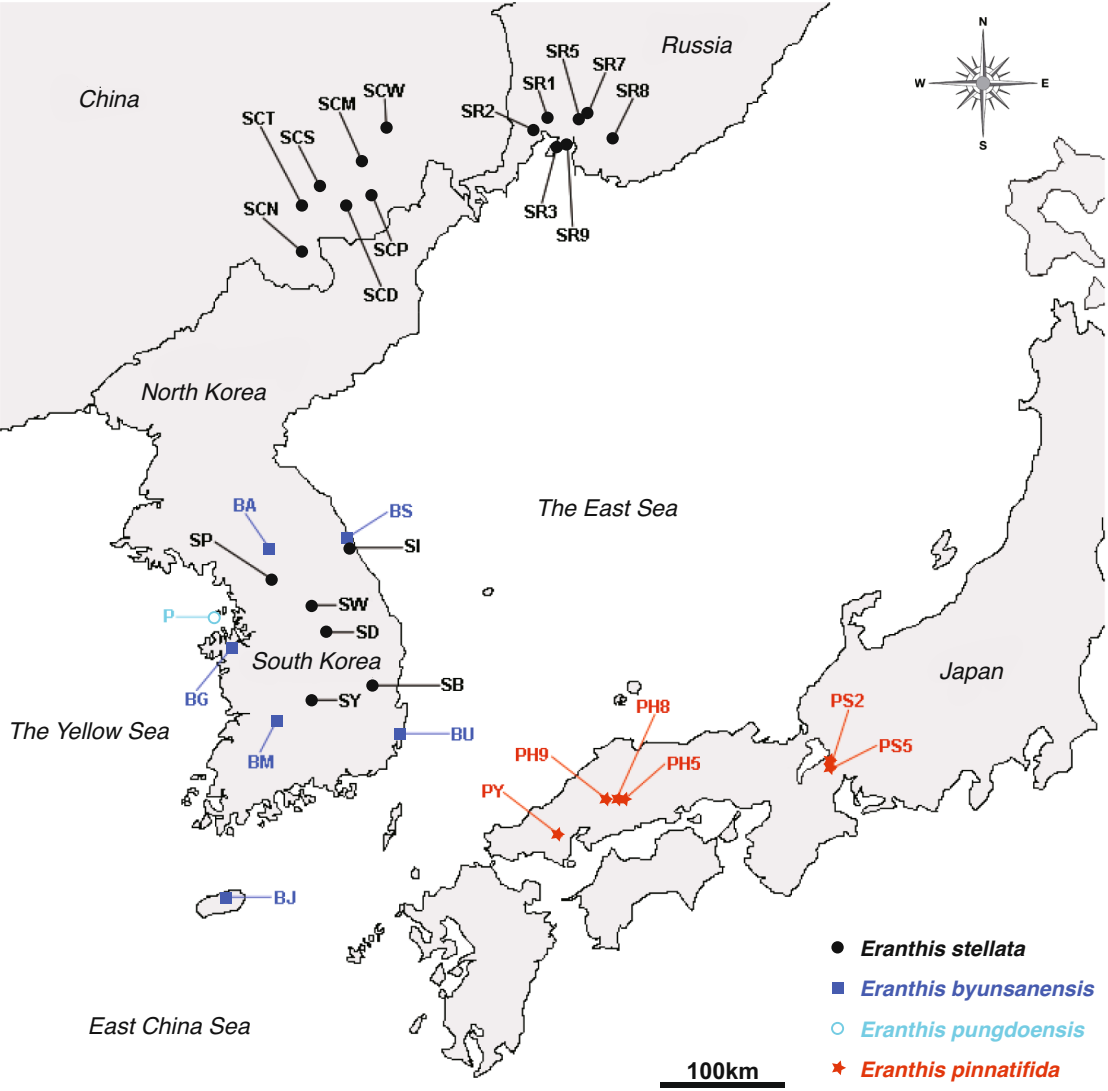
Collection sites of four *Eranthis* species; *E.* *stellata*, *E.* *byunsanensis*, *E.* *pungdoensis*, and *E.* *pinnatifida*. Black circles denote the material sampling sites for *E.* *stellata*; blue squares for *E.* *byunsanensis*; light blue empty circle for *E.* *pungdoensis*; and red stars for *E.* *pinnatifida*. Modified from Oh and Oh (2019)

Leaves or bracts were collected from a total of 935 individuals from the four studied *Eranthis* species, and approximately 30 individuals were sampled for each population. Specifically, 572 *E.* *stellata* individuals were collected from 20 populations in Korea, China, and Russia, 162 *E.* *byunsanensis* individuals were collected from 6 populations in South Korea, 30 *E.* *pungdoensis* individuals were collected from 1 population in the island Pungdo, South Korea, and 171 *E.* *pinnatifida* individuals were collected from 6 populations in Honshu, Japan (Figure 2; Table 1). Due to the morphological and genetic similarity of *E.* *byunsanensis* and *E.* *pungdoensis*, these two species were analyzed together as if they belonged to the same species. During sampling, the distance between the neighboring individuals collected was set to approximately 1 m, although this distance varied depending on the sizes of the sampled populations. The number of sampled populations for each species generally reflects the total number of populations of each species.

**TABLE 1:** Information on the populations of *Eranthis* species used in this study (Oh & Oh, 2019)

Species	Sampling locations	Geographical coordinates	Altitude (m)	Code
*E. byunsanensis*	Jinan, Korea	N 35°45′38.0″ E 127°24′51.7″	503	BM
	Ulsan, Korea	N 35°34′39.2″ E 129°25′24.1″	96	BU
	Gapyeong, Korea	N 37°55′04.7″ E 127°25′07.1″	753	BA
	Jeju, Korea	N 33°26′10.4″ E 126°37′42.4″	576	BJ
	Sokcho, Korea	N 38°10′29.8″ E 128°29′07.4″	225	BS
	Yesan, Korea	N 36°42′41.3″ E 126°37′02.6″	240	BG
*E. pungdoensis*	Pungdo Is, Ansan, Korea	N 37°06′36.9″ E 126°23′11.9″	127	P
*E. stellata*	Wonju, Korea	N 37°15′36.6″ E 127°57′51.0″	682	SW
	Inje, Korea	N 38°02′35.3″ E 128°29′44.6″	944	SI
	Danyang, Korea	N 36°59′29.2″ E 128°15′56.4″	572	SD
	Namyangju, Korea	N 37°41′16.2″ E 127°15′09.1″	244	SP
	Yeongdong, Korea	N 36°02′00.9″ E 127°50′24.8″	881	SY
	Yeongcheon, Korea	N 36°10′17.3″ E 128°59′41.8″	758	SB
	Wangqing, Jilin, China	N 43°18′15.4″ E 129°18′53.4″	474	SCW
	Antu, Jilin, China–1	N 42°58′48.3″ E 128°41′22.4″	553	SCM
	Antu, Jilin, China–2	N 42°36′29.1″ E 128°00′52.6″	515	SCS
	Antu, Jilin, China–3	N 42°17′40.8″ E 127°49′15.9″	1096	SCT
	Fusong, Jilin, China	N 41°51′47.5″ E 127°41′51.3″	960	SCN
	Antu, Jilin, China–4	N 42°30′39.9″ E 128°30′58.5″	681	SCD
	Helong, Jilin, China	N 42°35′37.4″ E 128°54′20.5″	631	SCP
	Nadezhdinsky, Primorskiy kray, Russia	N 43°34′17.8″ E 131°51′10.6″	5	SR1
	Khasanskiy, Primorskiy kray, Russia	N 43°21′28.9″ E 131°39′15.7″	39	SR2
	Vladivostok, Primorskiy kray, Russia	N 43°12′42.4″ E 132°04′12.5″	81	SR3
	Shkotovskiy, Primorskiy kray, Russia–1	N 43°32′21.8″ E 132°25′07.4″	129	SR5
	Shkotovskiy, Primorskiy kray, Russia–2	N 43°35′21.7″ E 132°27′55.4″	162	SR7
	Partizansk, Primorskiy kray, Russia	N 43°20′34.1″ E 132°57′24.4″	514	SR8
	Gorod Artem, Primorskiy kray, Russia	N 43°16′30.1″ E 132°13′46.3″	45	SR9
*E. pinnatifida*	Inukami, Shiga Pref., Japan	N 35°14′34.3″ E 136°19′31.7″	345	PS2
	Higashi‐Ohmi‐shi, Shiga Pref., Japan	N 35°02′39.9″ E 136°19′26.4″	319	PS5
	Syobara‐shi, Hiroshima Pref., Japan	N 34°46′10.3″ E 133°06′20.6″	317	PH5
	Miyoshi‐shi, Hiroshima Pref., Japan–1	N 34°45′48.9″ E 132°59′48.8″	265	PH8
	Miyoshi‐shi, Hiroshima Pref., Japan–2	N 34°45′05.6″ E 132°47′49.6″	176	PH9
	Iwakuni‐shi, Yamaguchi Pref., Japan	N 34°15′19.6″ E 131°57′19.0″	138	PY

The collected leaves or bracts were dried with silica gel and voucher specimens were deposited in CBNU, the herbarium of Chungbuk National University in South Korea. DNA was extracted from 935 individuals of the four *Eranthis* species using the DNeasy Plant Mini kit (QIAGEN). Dried leaves or bracts of about 20 mg were crushed with Tissue Lyser (QIAGEN), and DNA extraction was conducted using RNase A and the buffers in the DNeasy Plant Mini kit. The extracted DNA was then diluted to a final concentration of 10–50 ng/µl for the downstream analyses.

### 2.2 Chloroplast microsatellite genotyping

Twelve chloroplast microsatellite (cpSSR) loci (Ebp01, Ebp40, Ebp27, Ebp31, Ebp25, Ebp12, Ebp10, Ebp06, Ebp38, Ebp11, Ebp28, and Ebp32) were genotyped in the 935 individuals of the four *Eranthis* species. These loci were randomly selected from 24 cpSSR loci isolated by Oh and Oh (2017).

PCR was conducted in a TAKARA PCR Thermal Cycler Dice Touch (TAKARA Bio Inc.) using the Multiplex Master Mix of the QIAGEN Multiplex Master kit (QIAGEN). The PCR amplification conditions and procedures were the same as those described by Oh and Oh (2017).

An ABI3730xl DNA Analyzer (Applied Biosystems) and GeneMapper v. 3.7 (Applied Biosystems) were then used to measure PCR product lengths.

In the previous phylogeographic study of the authors (Oh & Oh, 2019), the same genotype data for 935 individuals of the genus *Eranthis* were used to analyze genetic diversity, population structure, and other evolutionary parameters.

### 2.3 Data analyses

#### 2.3.1 Genetic differentiation

For each *Eranthis* species, genetic differentiation between populations was estimated using two parameters, F_ST_ (Wright, 1943, 1951, 1965) and R_ST_ (Slatkin, 1995), which is an analog of F_ST_. Arlequin 3.5 (Excoffier & Lischer, 2010) was used for this analysis.

We used both F_ST_ and R_ST_ because they both have their own drawbacks and we wanted these two measures to complement each other (Balloux and Lugon‐Moulin, 2002). For example, F_ST_ is known to be sensitive to mutation rates when migration is low (Balloux and Lugon‐Moulin, 2002), and this can pose problems when analyzing microsatellite data, which shows high mutation rate. In contrast, R_ST_, which is suitable for analyzing markers with the stepwise mutation model (SMM), is less sensitive to high mutation rates reported in microsatellite, thus complementing the weakness of the F_ST_ (Holsinger & Weir, 2009). Meanwhile, due to the high variance of R_ST_, F_ST_ may outperform R_ST_ even under strict SMM (Gaggiotti et al., 1999; Balloux and Lugon‐Moulin, 2002). In fact, there is no mutation model that perfectly fits the behavior of microsatellites, and therefore F_ST_ and R_ST_ are often used together in microsatellite data analyses (Ando et al., 2011; Balloux and Lugon‐Moulin, 2002; De March et al., 2002; Sander et al., 2018).

#### 2.3.2 Genetic distance

As an additional tool for understanding the genetic differences between the populations in this genus, genetic distance was measured so that this parameter can effectively complement the genetic differentiation analysis above. For each *Eranthis* species, genetic distances between populations were calculated using the Microsatellite Analyzer (MSA) 4.05 (Dieringer & Schlötterer, 2003).

Among the many genetic distance parameters available, (δµ)^2^, which was developed by Goldstein et al. (1995), was used for the analysis of microsatellite data. (δµ)^2^ was developed based on stepwise mutation model which can be successfully applied to microsatellite loci (Goldstein et al., 1995). (δµ)^2^ is known to be a linear function of the time since divergence between the populations (Goldstein et al., 1995). The values for (δµ)^2^ in this study provide an estimation of the evolutionary distances between the populations.

#### 2.3.3 Isolation by distance

Our study sought to determine if there were significant relationships between the genetic distances and geographic distances for each species. The Mantel test (Mantel, 1967), which is a broadly used statistical test to evaluate the association between distance matrices, was conducted to assess the correlation between genetic distance (F_ST_ in this study) and geographic distance. In other words, this test was conducted to identify whether we can find the pattern of isolation by distance in the data. The Mantel tests were conducted using GenAlEX 6.5 (Peakall & Smouse, 2012).

#### 2.3.4 Gene flow

The coalescence‐based MIGRATE‐N 3.6.11 (Beerli & Palczewski, 2010) was used to infer the level and pattern of historical gene flow in each species. In the case of *E.* *stellata*, the 20 populations that were analyzed together in the above analyses were divided into three groups according to their geographic regions (seven Russian populations, seven Chinese populations, and six South Korean populations). This measure was taken because analyzing all 20 populations in a single run would have been extremely time‐consuming, and even if the analysis itself was completed, there was a possibility that the results would not have been meaningful (personal communication with Peter Beerli, the author of MIGRATE‐N).

In the MIGRATE‐N analyses, mutation‐scaled effective population sizes (θ = Neµ; where Ne is the effective population size and µ is the mutation rate per site per generation) and mutation‐scaled immigration rates (M = m/µ; m is the immigration rate) were determined. The number of immigrant individuals per generation was calculated by multiplying θ by M. Bayesian inference was used in these analyses and the Brownian motion model was selected as the data type. The results of applying a Brownian motion model are very similar to those of applying a stepwise mutation model, but the time it takes to analyze is much shorter with the Brownian motion model. Other parameters in the MIGRATE‐N analysis were set to the default values or were established based on the suggestions provided by the MIGRATE Manual V. 3.2.1 (Beerli, 2012).

The relationship between migration rate and geographic distance was analyzed using a regression analysis tool in Excel 2013 to determine if the level of migration was affected by geographic distances between the populations in each species.

#### 2.3.5 Bottleneck

BOTTLENECK 1.2 (Piry et al., 1999) was used to detect recent decreases in population size for each population and explain the patterns of genetic differentiation and gene flow in the studied genus. Here, “recent” is defined as within the last 2–4 Ne generations. Given that our data contained less than 20 loci, Wilcoxon's test was utilized, as it is the most powerful and robust approach under this condition. Based on the manual, we selected a two‐phased model of mutation (TPM) with 95% single‐step mutations, 5% multiple‐step mutations, and a variance of 12, for our microsatellite data analysis.

## 3 RESULTS

### 3.1 Genetic differentiation and genetic distance

In the *E.* *byunsanensis* and *E.* *pungdoensis* populations, all the pairwise F_ST_ values were close to 1, ranging from 0.7632 to 0.95196 (Table 2). R_ST_ values also closely approached 1, ranging from 0.95861 to 0.99973 (Table 2). These high values are indicative of significant genetic differentiation between these populations. For *E.* *pinnatifida*, the F_ST_ values ranged from 0.01153 to 0.90254, and R_ST_ values ranged from 0.01153 to 0.9532 (Table 3). In general, *E.* *pinnatifida* exhibited lower F_ST_ and R_ST_ values than *E.* *byunsanensis* and *E.* *pungdoensis*. The genetic differentiation values between PH5 and PH8, and PH5 and PH9 were much lower than in other cases, and the differentiation between PH8 and PH9 was extremely low (F_ST_, R_ST_ = 0.01153; *p* = .33; Table 3). Except for the lowest value, all genetic differentiation values in *E.* *pinnatifida* were significant. In the *E.* *stellata* populations, F_ST_ values ranged from 0.19908 (0.2) to 0.93366 (0.93), and R_ST_ values ranged from 0.10817 (0.11) to 0.99894 (1) (Table 4). All these values in *E.* *stellata* were indicative of significant genetic differentiation.

**TABLE 2:** Matrix of population pairwise F_ST_ (below diagonal) and R_ST_ (above diagonal) in *E.* *byunsanensis* and *E.* *pungdoensis*. All values indicated significant population pairwise genetic differentiations

	BA	BG	BJ	BM	BS	BU	P
BA	0	0.99499	0.9997	0.97139	0.98769	0.9895	0.96859
BG	0.94735	0	0.99969	0.99301	0.99486	0.99613	0.99258
BJ	0.87441	0.89778	0	0.99964	0.99973	0.99964	0.99969
BM	0.90855	0.92333	0.7632	0	0.9829	0.98463	0.97926
BS	0.94713	0.95196	0.91051	0.92354	0	0.99228	0.95861
BU	0.90903	0.92972	0.80407	0.88943	0.93057	0	0.98985
P	0.92593	0.93614	0.87068	0.91076	0.89098	0.89788	0

**TABLE 3:** Matrix of population pairwise F_ST_ (below diagonal) and R_ST_ (above diagonal) in *E.* *pinnatifida*. Except for F_ST_ value and R_ST_ value between PH8 and PH9 (bold value = 0.01153; *p* = .33), all values indicated significant population pairwise genetic differentiations

	PH5	PH8	PH9	PS2	PS5	PY
PH5	0	0.5875	0.54853	0.88826	0.82509	0.84811
PH8	0.5875	0	**0.01153**	0.91239	0.8849	0.90064
PH9	0.54853	**0.01153**	0	0.90307	0.86743	0.88517
PS2	0.81945	0.86798	0.85372	0	0.74895	0.9532
PS5	0.82509	0.8849	0.86743	0.74895	0	0.9488
PY	0.84811	0.90064	0.88517	0.87418	0.90254	0

**TABLE 4:** Matrix of population pairwise F_ST_ (below diagonal) and R_ST_ (above diagonal) in *E.* *stellata*. All values indicated significant population pairwise genetic differentiations

	SR1	SR2	SR3	SR5	SR7	SR8	SR9	SCW	SCN	SCM	SCS	SCT	SCP	SCD	SY	SP	SD	SI	SW	SB
SR1	0	0.25	0.11	0.34	0.22	0.62	0.27	0.79	0.87	0.74	0.75	0.87	0.99	0.77	0.97	0.96	0.97	0.94	0.96	0.96
SR2	0.25	0	0.34	0.19	0.22	0.53	0.41	0.61	0.88	0.76	0.75	0.87	0.99	0.8	0.97	0.96	0.97	0.95	0.96	0.96
SR3	0.34	0.4	0	0.66	0.24	0.65	0.47	0.95	0.96	0.9	0.88	0.96	1	0.91	0.99	0.99	0.99	0.98	0.99	0.99
SR5	0.43	0.25	0.64	0	0.2	0.64	0.63	0.83	0.92	0.79	0.78	0.92	1	0.84	0.99	0.98	0.99	0.97	0.98	0.98
SR7	0.36	0.28	0.5	0.29	0	0.25	0.24	0.21	0.2	0.14	0.14	0.18	0.57	0.22	0.37	0.32	0.39	0.32	0.35	0.33
SR8	0.43	0.41	0.66	0.47	0.2	0	0.61	0.67	0.87	0.75	0.77	0.86	0.99	0.81	0.96	0.94	0.96	0.93	0.95	0.95
SR9	0.47	0.55	0.52	0.68	0.45	0.56	0	0.91	0.95	0.86	0.86	0.95	1	0.89	0.99	0.99	0.99	0.97	0.99	0.99
SCW	0.67	0.6	0.89	0.66	0.39	0.54	0.84	0	0.98	0.93	0.91	0.97	1	0.95	1	0.99	1	0.98	0.99	1
SCN	0.69	0.75	0.86	0.79	0.58	0.71	0.83	0.86	0	0.73	0.47	0.41	1	0.73	0.99	0.99	0.98	0.95	0.98	0.98
SCM	0.65	0.73	0.86	0.75	0.53	0.68	0.8	0.86	0.63	0	0.2	0.78	1	0.61	0.98	0.98	0.98	0.95	0.98	0.98
SCS	0.62	0.69	0.82	0.74	0.5	0.64	0.77	0.83	0.4	0.24	0	0.55	0.99	0.58	0.97	0.96	0.97	0.93	0.97	0.96
SCT	0.68	0.75	0.87	0.81	0.59	0.72	0.83	0.9	0.49	0.74	0.49	0	1	0.79	0.99	0.98	0.98	0.94	0.98	0.98
SCP	0.75	0.77	0.91	0.81	0.61	0.81	0.9	0.91	0.89	0.87	0.85	0.91	0	1	1	1	1	1	1	1
SCD	0.56	0.63	0.81	0.64	0.53	0.67	0.82	0.81	0.75	0.76	0.73	0.82	0.85	0	0.98	0.98	0.98	0.95	0.98	0.97
SY	0.71	0.71	0.89	0.75	0.57	0.73	0.88	0.87	0.82	0.85	0.79	0.87	0.91	0.84	0	0.98	0.99	0.98	0.99	1
SP	0.69	0.72	0.88	0.74	0.56	0.71	0.86	0.86	0.83	0.81	0.78	0.87	0.9	0.8	0.81	0	0.99	0.99	0.99	0.99
SD	0.69	0.76	0.87	0.79	0.67	0.79	0.87	0.91	0.75	0.77	0.71	0.83	0.9	0.73	0.87	0.87	0	0.86	0.94	0.95
SI	0.63	0.66	0.79	0.71	0.49	0.62	0.74	0.78	0.67	0.7	0.62	0.72	0.84	0.72	0.76	0.73	0.74	0	0.63	0.75
SW	0.73	0.76	0.89	0.81	0.6	0.73	0.85	0.9	0.79	0.81	0.76	0.86	0.92	0.82	0.88	0.84	0.85	0.49	0	0.89
SB	0.66	0.73	0.88	0.79	0.66	0.78	0.87	0.93	0.88	0.88	0.83	0.89	0.93	0.85	0.91	0.87	0.86	0.7	0.86	0

The genetic distance parameter (δµ)^2^ was estimated in the three groups defined above. In the *E.* *byunsanensis* and *E.* *pungdoensis* populations, the genetic distances between the BJ (Jeju island) and all the other populations were extremely high, ranging from 322.9612 to 347.0752 (Table S1). The other genetic distances in this group ranged from 1.03167 to 14.29879 (Table S1). In the case of *E.* *pinnatifida*, the genetic distance values between the PH5 and PH8, PH5 and PH9, and PH8 and PH9 populations were lower than 0.1 (Table S1). The lowest value, 0.00269, was observed between PH8 and PH9. The (δµ)^2^ value between PS2 and PS5 was 0.17599, which was rather low. In the other *E.* *pinnatifida* cases, the genetic distance values ranged from 0.25594 to 1.01348. In *E.* *stellata*, the lowest value of 0.05 was observed between the SR1 and SR3 populations (Table S1). The highest value of 55.89 was observed between the SCP and SW populations. Interestingly, the genetic distance values between SCP and all the other populations were extremely high (i.e., higher than 50; Table S1). This result was attributed to the genetic differences between the SCP population and the rest of the *E. stellata* populations, which was elucidated in a previous study (Oh & Oh, 2019), where the reason for this difference was not identified.

### 3.2 Isolation by distance

**FIGURE 3:**
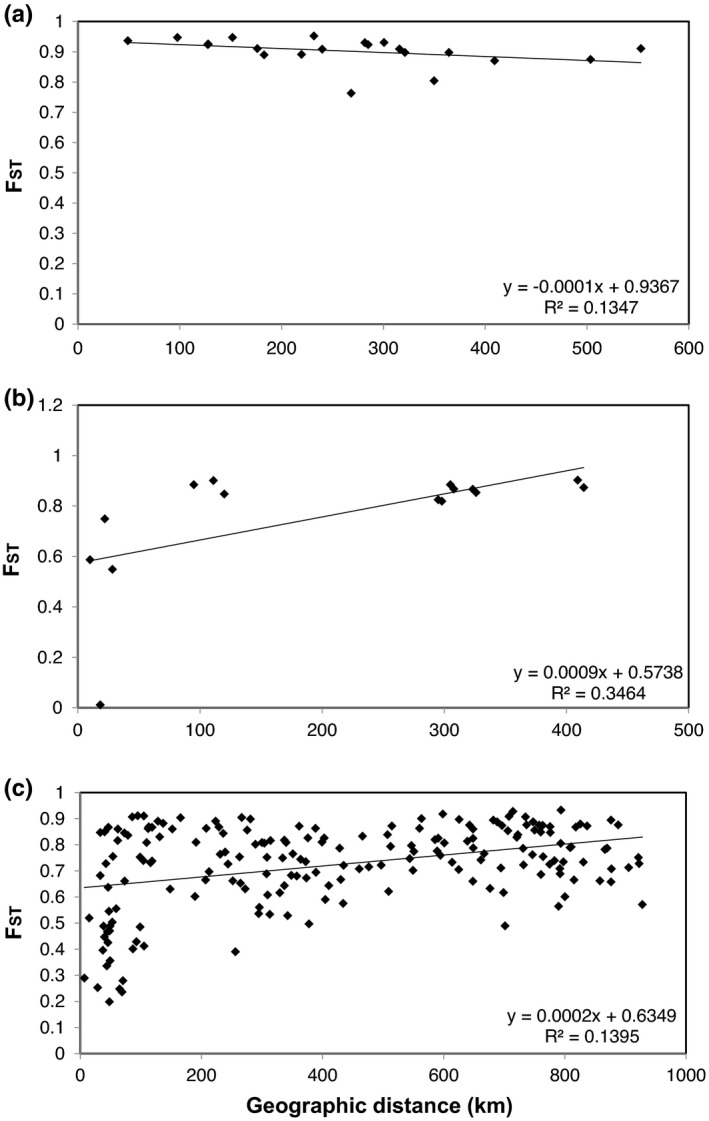
Relationship between geographic distance and genetic distance (F_ST_) for four *Eranthis* species. Scatter plots for (a) the populations of *E.* *byunsanensis* and *E.* *pungdoensis*, (b) the populations of *E.* *pinnatifida*, and (c) the populations of *E.* *stellata*

Each of the three groups identified above was analyzed to determine whether there was a clear relationship between genetic distance (in this analysis, F_ST_) and geographic distance in the focal species, and the Mantel test (Figure 3) was used here. The Mantel test identified a negative correlation in the *E.* *byunsanensis* and *E.* *pungdoensis* populations, which indicates that isolation by distance is not established in this group (*R^2^* = .1347, *p* = .16; Figure 3a). In *E.* *pinnatifida*, we observed a positive correlation between genetic distance and geographic distance, which indicates the existence of isolation by distance in these populations (*R* ^2^ = .3464, *p* = .05; Figure 3b). In the case of *E.* *stellata*, genetic distance increased with geographic distance, as observed in *E.* *pinnatifida*, and isolation by distance was also identified in these populations (*R* ^2^ = .1395, *p* = .01; Figure 3c). Taken together, our Mantel test results show that isolation by distance is generally established in this genus.

### 3.3 Historical gene flow

The long‐term effective population size (θ), the long‐term migration rate (M), and the number of migrants (Nm) were calculated in five groups (1. *E.* *byunsanensis* and *E.* *pungdoensis*, 2. *E.* *pinnatifida*, 3. Russian populations of *E.* *stellata*, 4. Chinese populations of *E.* *stellata*, and 5. Korean populations of *E.* *stellata*; Table 5). In these five groups, historical migration events appeared to have occurred in a complex manner, and it was difficult to extract specific patterns from these migration‐related data. In most cases, the migrations between the populations were asymmetrical.

**TABLE 5:** Mean migration rate (*M*) and mean number of migrants (Nm) for each of the five groups estimated from MIGRATE‐N

	Mean Migration rate (*M*)	Mean No. of migrants (Nm)
*Eranthis byunsanensis*/*Eranthis pungdoensis*	4.571	1.289
*Eranthis pinnatifida*	33.708	47.001
*Eranthis stellata*–Russia	8.430	4.582
*Eranthis stellata*–China	4.288	15.293
*Eranthis stellata*–Korea	14.739	5.959

Except for an outlier with a 65.494 value, the migration rates of the *E.* *byunsanensis* and *E.* *pungdoensis* populations ranged from 1.222 (95% CI: 0.0–3.5) to 16.079 (95% CI: 13.5–30.0; Table S2). The number of migrants ranged from 0.318483 to 4.233601, excluding the outlier value 17.75542 (Table S2). In these populations, the effective population sizes were comparable, ranging from 0.25914 to 0.32588 (Table S3).

In *E.* *pinnatifida*, the migration rates ranged from 4.126 (95% CI: 0.0–11.5) to 79.766 (95% CI: 56.5–98.0), and these values were much higher than those of the other groups (Table S2). The migrant numbers were also very high, with the highest value being 270.0269 (Table S2). In this species, the effective population sizes ranged from 0.57246 to 4.96072 (Table S3).

In Russian *E.* *stellata*, the migration rates ranged from 1.115 (95% CI: 0.0–3.0) to 46.117 (95% CI: 39.0–54.0), and the number of migrants ranged from 0.444702 to 31.136354 (Table S2). The values of θ ranged from 0.274 to 0.87245 (Table S3). In Chinese populations, the migration rates ranged from 0.997 (95% CI: 0.0–3.0) to 22.17 (95% CI: 0.5–6.0; Table S2). The values of θ were 0.28404–0.63414 (Table S3). In Korean populations, the migration rates ranged from 1.211 (95% CI: 0.0–3.0) to 60.863 (95% CI: 38.0–61.5) and the numbers of immigrants from 0.534233 to 21.42804 (Table S2). The values of θ were 0.27694–0.63863 (Table S3).

**FIGURE 4:**
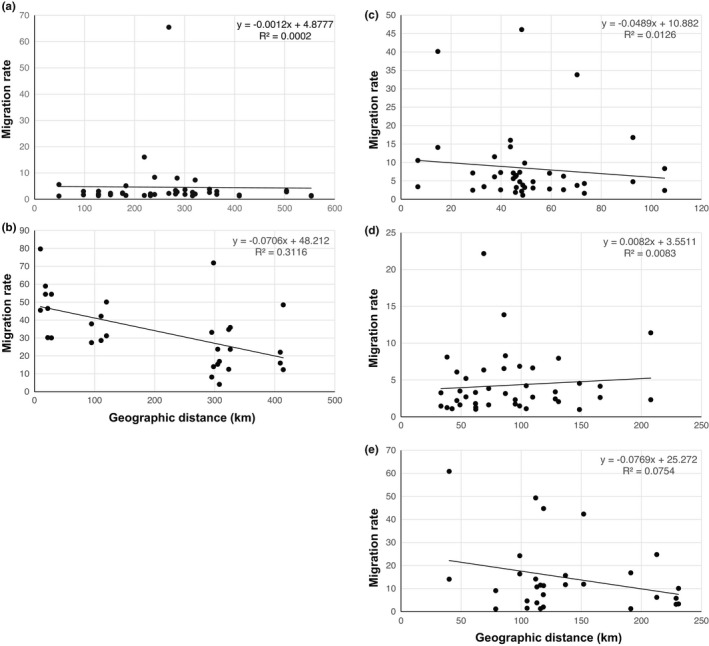
Relationship between migration rates and geographic distance for four *Eranthis* species. Scatter plots for (a) the populations of *E.* *byunsanensis* and *E.* *pungdoensis*; (b) the populations of *E.* *pinnatifida*; (c) Russian populations of *E.* *stellata*; (d) Chinese populations of *E.* *stellata*; and (e) Korean populations of *E.* *stellata*

The relationship between migration rate, which was derived from historical gene flow analyses, and geographic distance was investigated as well to obtain additional insights into the gene flow dynamics of this genus. Except for the Chinese *E.* *stellata* populations (Figure 4d), the four remaining groups exhibited negative correlations between migration rate and geographic distance (Figure 4a–c,e). This indicates that there existed a notable relationship between the degree of migration and the geographic distance in the focal species and that geographic distance markedly constrained successful migrations in these populations.

### 3.4 Bottleneck analysis

According to the BOTTLENECK test, a large number of the populations in the four species studied herein exhibited mode shifts in the allele frequency distributions, indicating that these populations have experienced a recent bottleneck.

In *E.* *byunsanensis* and *E.* *pungdoensis*, the BA, BM, BS, BU, and P populations experienced a recent bottleneck. In *E.* *pinnatifida*, PH5, PH9, and PS2 appeared to have experienced a recent reduction in population sizes. In *E.* *stellata*, the Russian populations SR1, SR2, SR7, and SR8, and the Chinese populations SCW, SCM, SCS, and SCD experienced bottlenecks. In the case of the South Korean *E.* *stellata*, most of the populations seem to have experienced bottlenecks (SY, SP, SD, SW, and SB, five of six populations).

## 4 DISCUSSION

### 4.1 Mechanisms of exceptionally high genetic differentiation in the four studied *Eranthis* species

In this study, the values of genetic differentiation measured using F_ST_ and R_ST_ were significant in all cases, with the exception of one *E. pinnatifida* instance (F_ST_, R_ST_ = 0.01153, *p* = .33; Table 3). A consensus on whether a certain F_ST_ value should be considered high or low has not been reached. However, in the studies of Hartl et al. (1997) and Frankham et al. (2002), it was suggested that F_ST_ values above 0.15 indicate significant differentiation. Using the aforementioned value as a reference, the genetic differentiation estimates in *E. byunsanensis* and *E. pungdoensis*, which approached a value of 1, were extremely high.

In a study by So et al. (2012), the genetic differentiation between five populations of *E. byunsanensis* was estimated using 10 allozyme markers, and the mean F_ST_ value was 0.131. This inconsistency between F_ST_ values in the So et al. study and our study can be partially explained by the genetic markers used in the analyses. Microsatellites are generally known to evolve much faster than allozyme loci, and better detect genetic differentiation between populations, with mutation rates of 10^−3^ to 10^−4^ (Dallas, 1992; Weber & Wong, 1993) compared to the 10^−6^ to 10^−9^ of the allozyme loci (Ayala, 1976). In previous studies on red pine (*Pinus resinosa*), no allozyme diversity was detected, whereas 23 haplotypes were recovered with 9 chloroplast microsatellite markers (Echt et al., 1998; Provan et al., 2001). Therefore, the high population differentiation values in our study likely derived from the high mutation rates of chloroplast microsatellites.

Genetic drift might have also influenced the high levels of genetic differentiation. In extreme cases, genetic drift can cause allele fixation in opposite directions in many populations, resulting in very high genetic differences between the populations (Jeong, 2010; Nistelberger et al., 2015). In smaller or isolated populations, genetic drift has an even stronger effect (Toczydlowski & Waller, 2019). In this study, the sampled populations were isolated and generally small, with only some populations being extremely large. This would have resulted in significant genetic drift in these populations. In the BOTTLENECK tests, we found that more than half of the studied populations had experienced recent bottlenecks. In the South Korean *E. stellata*, five of six populations experienced bottleneck events. These bottleneck events would have greatly facilitated genetic drift in these populations. Moreover, inbreeding and selfing might have promoted genetic drift by reducing the effective population size (Caballero & Hill, 1992; Lowe et al., 2005). Previous studies on *E. byunsanensis* (So et al., 2012) and *E. stellata* (Jeong et al., 2005) suggested that inbreeding is significantly occurring in these populations, which may ultimately contribute to genetic differentiation between the populations. However, additional studies on inbreeding in the genus *Eranthis* should be conducted to reach more reliable conclusions.

Finally, the high genetic differentiation observed herein might have also been due to selection. Selection can genetically structure populations when organisms are selectively adapted to gradient environments, which is also referred to as isolation by environment or isolation by adaptation (Nosil et al., 2008; Sexton et al., 2014; Wang & Summers, 2010). For example, the *E. byunsanensis* and *E. pungdoensis* populations are geographically separated from the others (>150 km), and different selective pressures likely acted in the different locations where these populations were located, resulting in different genetic characteristics between the populations. Even though morphological differences are not clearly observed between these widely distributed populations, adaptive genotypes might have still been generated, resulting in high genetic differentiation. However, in our study, selection as an important factor affecting genetic differentiation is not yet supported by actual analyses or observations, and further research is needed to define the extent to which the high genetic differentiation observed is due to selection. Overall, the high genetic differentiation values in our study are likely due to a combination of all of the aforementioned factors, which are the genetic marker used, genetic drift, and possibly selection.

### 4.2 Unexpected correlation between genetic distance and geographic distance in the genus *Eranthis*

As mentioned above, we initially hypothesized that geographic distances between populations would not meaningfully affect genetic distances in the absence of gene flow considering that gene flow would be the major factor which can generate the correlation between geographic distance and genetic distance. However, our results support that the values of F_ST_, R_ST_, and (δµ)^2^, which indicate that genetic distances are clearly correlated with geographic distances in the absence of current gene flow.

In the case of *E. byunsanensis* and *E. pungdoensis*, the values of F_ST_, R_ST_, (δµ)^2^, and the Mantel test results indicate that genetic differentiation does not reflect geographic distance, which appeared to support our initial hypothesis.

However, in *E. pinnatifida* and *E. stellata*, a clear correlation was observed between genetic differentiation and geographic distance. For example, in *E. pinnatifida*, Hiroshima populations PH5, PH8, and PH9, which are located close to the others (10–30 km), exhibited significantly low genetic differentiation (F_ST_ and R_ST_) and genetic distance (δµ)^2^. Similarly, two Shiga populations of this species where the geographic distance was approximately 20 km also exhibited a notably low genetic distance.

Overall, as discussed above, genetic differentiation and genetic distance appeared to reflect the geographic distance in many cases within the genus *Eranthis*. However, additional studies are required to identify the mechanisms by which genetic differentiation reflects geographic distance in *Eranthis* populations in cases where gene flow between populations seems to be impossible. Here, we propose two different hypotheses that could explain the counterintuitive correlations between geographic distance and genetic distance (genetic differentiation) observed herein.

First, it is probable that gene flow historically existed, and this past event determined the current correlation between genetic differentiation and geographic distance. Our historical gene flow analysis using MIGRATE‐N demonstrates that there existed substantial gene flow long time ago, supporting this hypothesis. The occurrence of past gene flow means that geographic distance had previously affected gene flow (the correlation between migration rate and geographic distance was verified in our study; Figure 4), and therefore genetic distance still reflects geographic distance. Here, regarding the high genetic differentiation observed and the low possibility of current gene flow, which seem to be inconsistent with past gene flow, we suggest that historical gene flow and current gene flow may not have identical effects on genetic differentiation, even though this can be a rather bold argument and need further investigations to prove it. There are previous studies which tried to characterize both current gene flow and past gene flow at the same time in the target systems, and their results implicate that historical gene flow and current gene flow can be separately analyzed and approached from different perspectives (Chauvet et al., 2004; Lada et al., 2008).

In fact, it would be challenging to develop a specific model of historical gene flow in some *Eranthis* species given the observed current unlikeliness of gene flow due to the long distances between the populations. However, it is possible that *Eranthis* populations were both larger and more abundant in the past. This indicates that the geographic distances between the populations were shorter than they are now, which might have facilitated gene flow at that time. In our research, bottlenecks were found to have recently occurred in a large number of studied populations, supporting past prevalent population size reductions inferred above. The frequent occurrence of bottlenecks observed in this study indicates that the population size can be easily reduced in this taxon. Additionally, we also speculate that two or more relatively close present populations might have belonged to one very large population in the past, thus allowing for genetic interactions within the population (In relation to this suggestion, it is well established that population fragmentation reduces gene flow (Browne & Karubian, 2018; Couvet, 2002)). The current existence of very large populations supports this hypothesis as a possible explanation. To build a more concrete hypothesis on past gene flow, all current populations should be identified and analyzed and more populations should be included in future population genetic studies of this genus.

Secondly, we suggest another hypothesis where these *Eranthis* species did not experience gene flow in the past. At some time point in the history, the *Eranthis* populations might have reached specific distribution patterns that were not very different from those observed today, without having experienced gene flow. After that, selective forces might have acted on widely distributed populations for a long time, with each population adapting to its own environment, including the local climate (temperature, precipitation, etc.), soil properties, and other abiotic / biotic conditions. Generally, populations that are separated by long distances tend to inhabit substantially different environments and experience different evolutionary pressures compared to populations in closer proximity. This correlation between geographic distance and environmental difference is well established (Lee & Mitchell‐Olds, 2011; Wang & Bradburd, 2014). Therefore, the distance between the populations might have affected genetic differentiation via adaptation. Additionally, it is also likely that different mutations occurred with time within each population, thus further contributing to the genetic differences between the populations (Slatkin, 1987). These two hypotheses proposed herein should be further refined and tested to explain the intriguing and counterintuitive population genetic phenomena observed in this genus.

## 5 CONCLUSION

In this study, we observed interesting phenomena regarding genetic differentiation and gene flow in the genus *Eranthis*, and provided preliminary insights into the population genetics and dynamics of this genus. The extremely high genetic differentiation observed herein is likely due to a combination of the genetic markers used to conduct our analyses, genetic drift, and possibly selection. Unexpected positive correlations were identified between genetic distance and geographic distance in the absence of gene flow and two hypotheses were proposed to explain these observations. The current correlation between genetic distance and geographic distance may have resulted from past gene flow, or the action of adaptation to different environments in different locations. It is also probable that both phenomena acted in combination. We recognize that there can be further reasonable explanations for our observations and that future studies should incorporate all of the existing populations of these species to better characterize their population dynamics. In conclusion, our research provides an important case study that addressed intriguing and fundamental population genetic questions, and establishes a precedence for the analysis of population dynamics in the genus *Eranthis*, a lesser‐studied wild plant. In addition, our findings indicate that this genus can provide important and unique insights into the mechanisms of genetic differentiation and gene flow, and could therefore serve as a valuable system to facilitate the understanding of unexplored population genetics phenomena.

## AUTHOR CONTRIBUTIONS

**Ami Oh:** Conceptualization (equal); Data curation (lead); Formal analysis (lead); Investigation (lead); Writing – original draft (lead). **Byoung‐Un Oh:** Conceptualization (equal).

## CONFLICT OF INTEREST

The authors declare no conflict of interest.

## Supporting information

Table S1

Table S2

Table S3

## Data Availability

The data presented in this study has been uploaded to Dryad (https://doi.org/10.5061/dryad.w9ghx3frr).
