# Supplementary material for: Genetic differentiation that is exceptionally high and unexpectedly sensitive to geographic distance in the absence of gene flow: Insights from the genus Eranthis in East Asian regions
Source: Ecol Evol. 2022 Jun 7;12(6):e9007. doi: 10.1002/ece3.9007 (PMC9173865; doi:10.1002/ece3.9007)
Supplement: Supplementary file 1 — Table S1 [file ECE3-12-e9007-s003.docx]

**Oh – *Ecology and Evolution***

**Table S1**. Genetic distance between populations (dµ)^2^ in (A) *E. byunsanensis* and *E. pungdoensis*, (B) *E. pinnatifida*, and (C) *E. stellata*.

**(A)**

|  | **BA** | **BG** | **BJ** | **BM** | **BS** | **BU** | **P** |
| --- | --- | --- | --- | --- | --- | --- | --- |
| **BA** | 0 |  |  |  |  |  |  |
| **BG** | 7.48257 | 0 |  |  |  |  |  |
| **BJ** | 322.9612 | 319.866 | 0 |  |  |  |  |
| **BM** | 2.15806 | 8.52473 | 333.4776 | 0 |  |  |  |
| **BS** | 3.00557 | 6.06969 | 345.416 | 3.34917 | 0 |  |  |
| **BU** | 5.64178 | 14.29879 | 347.0752 | 5.29521 | 6.92561 | 0 |  |
| **P** | 1.54333 | 6.05123 | 345.2018 | 3.32583 | 1.03167 | 6.58789 | 0 |

**(B)**

|  | **PH5** | **PH8** | **PH9** | **PS2** | **PS5** | **PY** |
| --- | --- | --- | --- | --- | --- | --- |
| **PH5** | 0 |  |  |  |  |  |
| **PH8** | 0.08907 | 0 |  |  |  |  |
| **PH9** | 0.08454 | 0.00269 | 0 |  |  |  |
| **PS2** | 0.58515 | 0.68029 | 0.6717 | 0 |  |  |
| **PS5** | 0.25594 | 0.34512 | 0.34344 | 0.17599 | 0 |  |
| **PY** | 0.2577 | 0.34287 | 0.34386 | 1.01348 | 0.50079 | 0 |

|  | **SR1** | **SR2** | **SR3** | **SR5** | **SR7** | **SR8** | **SR9** | **SCW** | **SCN** | **SCM** | **SCS** | **SCT** | **SCP** | **SCD** | **SY** | **SP** | **SD** | **SI** | **SW** | **SB** |
| --- | --- | --- | --- | --- | --- | --- | --- | --- | --- | --- | --- | --- | --- | --- | --- | --- | --- | --- | --- | --- |
| **SR1** | 0 |  |  |  |  |  |  |  |  |  |  |  |  |  |  |  |  |  |  |  |
| **SR2** | 0.21 | 0 |  |  |  |  |  |  |  |  |  |  |  |  |  |  |  |  |  |  |
| **SR3** | 0.05 | 0.18 | 0 |  |  |  |  |  |  |  |  |  |  |  |  |  |  |  |  |  |
| **SR5** | 0.21 | 0.1 | 0.27 | 0 |  |  |  |  |  |  |  |  |  |  |  |  |  |  |  |  |
| **SR7** | 6.71 | 6.59 | 7.14 | 6.01 | 0 |  |  |  |  |  |  |  |  |  |  |  |  |  |  |  |
| **SR8** | 1.54 | 1.08 | 1.29 | 1.36 | 8.14 | 0 |  |  |  |  |  |  |  |  |  |  |  |  |  |  |
| **SR9** | 0.14 | 0.26 | 0.1 | 0.31 | 7.2 | 1.17 | 0 |  |  |  |  |  |  |  |  |  |  |  |  |  |
| **SCW** | 1.16 | 0.49 | 1.14 | 0.63 | 6.18 | 1.35 | 1.03 | 0 |  |  |  |  |  |  |  |  |  |  |  |  |
| **SCN** | 2.42 | 2.6 | 2.68 | 2.02 | 5.99 | 4.82 | 2.64 | 3.61 | 0 |  |  |  |  |  |  |  |  |  |  |  |
| **SCM** | 1.4 | 1.54 | 1.6 | 0.98 | 5.45 | 2.88 | 1.42 | 2.28 | 0.58 | 0 |  |  |  |  |  |  |  |  |  |  |
| **SCS** | 1.7 | 1.71 | 1.88 | 1.21 | 5.25 | 3.29 | 1.77 | 2.39 | 0.26 | 0.13 | 0 |  |  |  |  |  |  |  |  |  |
| **SCT** | 2.49 | 2.47 | 2.74 | 1.97 | 5.83 | 4.89 | 2.72 | 3.22 | 0.1 | 0.83 | 0.38 | 0 |  |  |  |  |  |  |  |  |
| **SCP** | 49.8 | 49.91 | 50.34 | 49.45 | 29.63 | 53.58 | 50.35 | 49.94 | 47.79 | 47.72 | 47.4 | 47.48 | 0 |  |  |  |  |  |  |  |
| **SCD** | 1.38 | 1.71 | 1.65 | 1.19 | 6.67 | 3.41 | 1.71 | 3.04 | 0.54 | 0.47 | 0.52 | 0.79 | 49.55 | 0 |  |  |  |  |  |  |
| **SY** | 11.42 | 12.08 | 12.18 | 11.06 | 13.01 | 18.17 | 11.47 | 11.85 | 7.62 | 9.14 | 8.59 | 7.24 | 52.07 | 10.23 | 0 |  |  |  |  |  |
| **SP** | 8.26 | 7.57 | 8.62 | 7.27 | 10.55 | 11.74 | 7.86 | 5.87 | 6.85 | 7.31 | 6.73 | 5.87 | 50.17 | 8.99 | 3.14 | 0 |  |  |  |  |
| **SD** | 9.42 | 10.71 | 9.85 | 10.23 | 13.85 | 16.16 | 10.37 | 13.38 | 5.85 | 9.05 | 7.99 | 5.67 | 54.34 | 7.39 | 8.85 | 12.59 | 0 |  |  |  |
| **SI** | 6.56 | 7.32 | 6.77 | 7 | 10.56 | 10.89 | 7.12 | 9.38 | 3.48 | 6.03 | 5.03 | 3.33 | 54.63 | 4.82 | 9.41 | 10.94 | 1.06 | 0 |  |  |
| **SW** | 7.87 | 8.67 | 8.02 | 8.55 | 12 | 12.42 | 8.45 | 10.8 | 5.31 | 8.11 | 6.98 | 5.01 | 55.89 | 6.77 | 10.93 | 12.24 | 1.1 | 0.27 | 0 |  |
| **SB** | 7.11 | 7.55 | 7.33 | 7.35 | 10.71 | 11.67 | 7.85 | 9.29 | 4.34 | 7.08 | 5.91 | 3.84 | 54.02 | 5.78 | 9.99 | 10.4 | 1.2 | 0.44 | 0.41 | 0 |

**(C)**
