# Supplementary material for: Genetic differentiation that is exceptionally high and unexpectedly sensitive to geographic distance in the absence of gene flow: Insights from the genus Eranthis in East Asian regions
Source: Ecol Evol. 2022 Jun 7;12(6):e9007. doi: 10.1002/ece3.9007 (PMC9173865; doi:10.1002/ece3.9007)
Supplement: Supplementary file 2 — Table S2 [file ECE3-12-e9007-s002.docx]

**Oh – *Ecology and Evolution***

**Table S2**. The long term migration rates (*M*) and the number of migrants (Nm) for four *Eranthis* species from MIGRATE-N.

| **Population pair** | **Migration rate (*M*)** | | **No. of migrants (Nm)** | |
| --- | --- | --- | --- | --- |
|  | **Direction of migration (⇨)** | **Direction of migration (⇦)** | **Direction of migration (⇨)** | **Direction of migration (⇦)** |
| ***Eranthis byunsanensis, Eranthis pungdoensis*** | | | | |
| BA/BG | 2.269 | 1.695 | 0.627787 | 0.50611 |
| BA/BJ | 2.825 | 3.268 | 0.765858 | 0.975792 |
| BA/BM | 8.376 | 1.821 | 2.729571 | 0.543732 |
| BA/BS | 1.712 | 3.009 | 0.45077 | 0.898457 |
| BA/BU | 2.671 | 1.356 | 0.784873 | 0.404888 |
| BA/P | 3.062 | 1.823 | 0.793487 | 0.54433 |
| BG/BJ | 3.006 | 1.876 | 0.814927 | 0.519052 |
| BG/BM | 1.968 | 1.358 | 0.641332 | 0.375731 |
| BG/BS | 2.044 | 1.357 | 0.538185 | 0.375455 |
| BG/BU | 2.023 | 3.369 | 0.594459 | 0.932135 |
| BG/P | 1.229 | 5.631 | 0.318483 | 1.557985 |
| BJ/BM | 2.241 | 65.494 | 0.730297 | 17.75542 |
| BJ/BS | 1.222 | 1.418 | 0.321753 | 0.38442 |
| BJ/BU | 2.592 | 3.836 | 0.761659 | 1.03994 |
| BJ/P | 1.304 | 1.619 | 0.337919 | 0.438911 |
| BM/BS | 2.768 | 8.085 | 0.728814 | 2.63474 |
| BM/BU | 1.459 | 5.144 | 0.428727 | 1.676327 |
| BM/P | 2.416 | 2.211 | 0.626082 | 0.720521 |
| BS/BU | 3.635 | 1.874 | 1.068145 | 0.493424 |
| BS/P | 1.452 | 16.079 | 0.376271 | 4.233601 |
| BU/P | 2.021 | 7.35 | 0.523722 | 2.159798 |
| **MEAN** | **4.571** | | **1.289** | |
| ***Eranthis pinnatifida*** | | | | |
| PH5/PH8 | 79.766 | 45.521 | 68.47512 | 225.8169 |
| PH5/PH9 | 30.127 | 54.433 | 18.6685 | 270.0269 |
| PH5/PS2 | 71.946 | 14.051 | 41.18621 | 69.70308 |
| PH5/PS5 | 33.203 | 8.248 | 24.80663 | 40.91602 |
| PH5/PY | 50.084 | 31.236 | 52.55665 | 154.953 |
| PH8/PH9 | 58.994 | 54.458 | 36.55622 | 46.74947 |
| PH8/PS2 | 16.933 | 4.126 | 9.693465 | 3.541965 |
| PH8/PS5 | 23.761 | 15.388 | 17.75232 | 13.20983 |
| PH8/PY | 28.663 | 42.204 | 30.07809 | 36.23002 |
| PH9/PS2 | 35.934 | 23.682 | 20.57078 | 14.67479 |
| PH9/PS5 | 34.792 | 12.576 | 25.9938 | 7.792844 |
| PH9/PY | 37.886 | 27.447 | 39.75643 | 17.00781 |
| PS2/PS5 | 30.228 | 46.546 | 22.58394 | 26.64572 |
| PS2/PY | 12.392 | 48.485 | 13.00379 | 27.75572 |
| PS5/PY | 16.012 | 22.115 | 16.80251 | 16.52256 |
| **MEAN** | **33.708** | | **47.001** | |
| ***Eranthis stellata* - Russia** | | | | |
| SR1/SR2 | 2.466 | 7.141 | 0.8103523 | 6.207314 |
| SR1/SR3 | 14.268 | 16.054 | 3.909432 | 13.95494 |
| SR1/SR5 | 1.904 | 6.191 | 1.6611448 | 5.381527 |
| SR1/SR7 | 3.233 | 9.836 | 2.117615 | 8.549943 |
| SR1/SR8 | 16.78 | 4.776 | 11.329185 | 4.151538 |
| SR1/SR9 | 7.142 | 5.614 | 3.175476 | 4.87997 |
| SR2/SR3 | 11.58 | 6.094 | 3.17292 | 2.002549 |
| SR2/SR5 | 2.62 | 6.274 | 2.285819 | 2.061699 |
| SR2/SR7 | 3.773 | 33.838 | 2.471315 | 11.11951 |
| SR2/SR8 | 8.349 | 2.4 | 5.6369108 | 0.788664 |
| SR2/SR9 | 4.781 | 7.33 | 2.1257282 | 2.408711 |
| SR3/SR5 | 3.22 | 6.474 | 2.809289 | 1.773876 |
| SR3/SR7 | 4.774 | 3.043 | 3.12697 | 0.833782 |
| SR3/SR8 | 4.281 | 1.623 | 2.89036 | 0.444702 |
| SR3/SR9 | 14.107 | 40.171 | 6.2722543 | 11.00685 |
| SR5/SR7 | 10.572 | 3.43 | 6.92466 | 2.992504 |
| SR5/SR8 | 1.115 | 3.886 | 0.7528034 | 3.390341 |
| SR5/SR9 | 3.43 | 3.441 | 1.5250466 | 3.0021 |
| SR7/SR8 | 46.117 | 2.181 | 31.136354 | 1.428555 |
| SR7/SR9 | 2.568 | 7.289 | 1.1417842 | 4.774295 |
| SR8/SR9 | 2.802 | 7.08 | 1.2458252 | 4.780133 |
| **MEAN** | **8.430** | | **4.582** | |
| ***Eranthis stellata* - China** | | | | |
| SCW/SCN | 11.42 | 2.321 | 5.77989 | 0.659257 |
| SCW/SCM | 1.02 | 1.286 | 0.646823 | 0.365275 |
| SCW/SCS | 7.955 | 2.068 | 90.4497 | 0.587395 |
| SCW/SCT | 2.632 | 4.159 | 0.972208 | 1.181322 |
| SCW/SCP | 13.867 | 6.563 | 5.232158 | 1.864155 |
| SCW/SCD | 2.686 | 6.639 | 0.991752 | 1.885742 |
| SCN/SCM | 4.558 | 0.997 | 2.89041 | 0.504602 |
| SCN/SCS | 8.292 | 3.17 | 94.28145 | 1.6044 |
| SCN/SCT | 3.516 | 1.628 | 1.29874 | 0.823963 |
| SCN/SCP | 2.432 | 3.399 | 0.917618 | 1.720302 |
| SCN/SCD | 1.475 | 6.879 | 0.544614 | 3.481599 |
| SCM/SCS | 22.17 | 6.371 | 252.0767 | 4.040106 |
| SCM/SCT | 1.107 | 4.226 | 0.408904 | 2.679876 |
| SCM/SCP | 2.214 | 6.093 | 0.835364 | 3.863815 |
| SCM/SCD | 2.723 | 5.223 | 1.005413 | 3.312113 |
| SCS/SCT | 1.252 | 8.117 | 0.462464 | 92.29167 |
| SCS/SCP | 1.626 | 3.857 | 0.613506 | 43.85475 |
| SCS/SCD | 1.113 | 1.106 | 0.410953 | 12.57541 |
| SCT/SCP | 1.734 | 2.32 | 0.654256 | 0.856962 |
| SCT/SCD | 3.322 | 1.814 | 1.226582 | 0.670055 |
| SCP/SCD | 1.466 | 3.271 | 0.541291 | 1.234181 |
| **MEAN** | **4.288** | | **15.293** | |
| ***Eranthis stellata* - Korea** | | | | |
| SY/SP | 1.291 | 16.846 | 0.569525 | 4.665331 |
| SY/SD | 3.864 | 10.721 | 1.360398 | 2.969074 |
| SY/SI | 10.13 | 3.4 | 6.469322 | 0.941596 |
| SY/SW | 11.724 | 15.728 | 4.279143 | 4.355712 |
| SY/SB | 1.504 | 4.696 | 0.665385 | 1.30051 |
| SP/SD | 7.387 | 2.016 | 2.600741 | 0.889358 |
| SP/SI | 11.504 | 1.328 | 7.3468 | 0.585847 |
| SP/SW | 9.142 | 1.211 | 3.336739 | 0.534233 |
| SP/SB | 5.817 | 3.211 | 2.573499 | 1.416533 |
| SD/SI | 11.311 | 44.751 | 7.223544 | 15.75548 |
| SD/SW | 14.093 | 60.863 | 5.143804 | 21.42804 |
| SD/SB | 14.182 | 49.389 | 6.274259 | 17.38839 |
| SI/SW | 24.279 | 16.393 | 8.861592 | 10.46906 |
| SI/SB | 6.247 | 24.808 | 2.763735 | 15.84313 |
| SW/SB | 11.961 | 42.385 | 5.291666 | 15.4701 |
| **MEAN** | **14.739** | | **5.959** | |

Direction of migration(**⇨**) means that the migration occurred from left to right population in each population pair.
