# Supplementary material for: Genetic differentiation that is exceptionally high and unexpectedly sensitive to geographic distance in the absence of gene flow: Insights from the genus Eranthis in East Asian regions
Source: Ecol Evol. 2022 Jun 7;12(6):e9007. doi: 10.1002/ece3.9007 (PMC9173865; doi:10.1002/ece3.9007)
Supplement: Supplementary file 3 — Table S3 [file ECE3-12-e9007-s001.docx]

**Oh – *Ecology and Evolution***

**Table S3**. The long term effective population sizes (θ) for four *Eranthis* species from MIGRATE-N.

| ***E. byunsanensis* /**  ***E. pungdoensis*** | | | ***E. pinnatifida*** | | ***E. stellata*** | | | | | |
| --- | --- | --- | --- | --- | --- | --- | --- | --- | --- | --- |
|  |  |  |  |  | **Russian populations** | | **Chinese populations** | | **Korean population** | |
|  | θ |  | | θ |  | θ |  | θ |  | θ |
| BA | 0.29859 | PH5 | | 4.96072 | SR1 | 0.86925 | SCW | 0.28404 | SY | 0.27694 |
| BG | 0.27668 | PH8 | | 0.85845 | SR2 | 0.32861 | SCN | 0.50612 | SP | 0.44115 |
| BJ | 0.2711 | PH9 | | 0.61966 | SR3 | 0.274 | SCM | 0.63414 | SD | 0.35207 |
| BM | 0.32588 | PS2 | | 0.57246 | SR5 | 0.87245 | SCS | 11.37017 | SI | 0.63863 |
| BS | 0.2633 | PS5 | | 0.74712 | SR7 | 0.655 | SCT | 0.36938 | SW | 0.36499 |
| BU | 0.29385 | PY | | 1.04937 | SR8 | 0.67516 | SCP | 0.37731 | SB | 0.44241 |
| P | 0.25914 |  | |  | SR9 | 0.44462 | SCD | 0.36923 |  |  |
